# Resveratrol in Hepatitis C Patients Treated with Pegylated-Interferon-α-2b and Ribavirin Reduces Sleep Disturbance

**DOI:** 10.3390/nu9080897

**Published:** 2017-08-18

**Authors:** Manuela Pennisi, Gaetano Bertino, Caterina Gagliano, Michele Malaguarnera, Rita Bella, Antonio Maria Borzì, Roberto Madeddu, Filippo Drago, Giulia Malaguarnera

**Affiliations:** 1 Spinal Ospedale Cannizzaro, University of Catania, 95100 Catania, Italy; manuelapennisi78@gmail.com; 2 Department of Internal Medicine and Systemic Diseases, University of Catania, 95123 Catania, Italy; gaetanobertinounict@gmail.com; 3 Research Center “The Great Senescence”, University of Catania, 95100 Catania, Italy; caterinagagliano@nestweb.it (C.G.); michele.malaguarnera@gmail.com (M.M.); antoniomaria.borzi@gmail.com (A.M.B.); 4 Neurovisual Science Technology (NEST), SpinLab, 95100 Catania, Italy; 5 Department “G.F. Ingrassia”, Section of Neurosciences, University of Catania, 95123 Catania, Italy; rbella@unict.it; 6 Department of Biomedical Science, University of Sassari, 07100 Sassari, Italy; rmadeddu@uniss.it; 7 Department of Biomedical and Biotechnological Sciences, University of Catania, 95123 Catania, Italy; f.drago@unict.it

**Keywords:** resveratrol, interferons, ribavirin, hepatitis C, sleep disorders

## Abstract

Background: Hepatitis C virus infection and interferon treatment have shown to be risk factors for sleep disorder health-related quality of life. Aim: To determine whether the effects of resveratrol on sleep disorders were associated with different tests in subjects with chronic hepatitis C treated with Peg-IFN-α and RBV. Patients and Methods: In this prospective, randomized, placebo controlled, double blind clinical trial, 30 subjects (Group A) with chronic hepatitis received Pegylated-Interferon-α2b (1.5 mg/kg per week), Ribavirin and placebo (*N*-acetylcysteine 600 mg and lactoferrin 23.6 g), while 30 subjects (Group B) received the same dosage of Pegylated-Interferon-α2b, Ribavirin and association of *N*-acetylcysteine 600 mg, lactoferrin 23.6 g and Resveratrol 19.8 mg for 12 months. All subjects underwent laboratory exams and questionnaires to evaluate mood and sleep disorders (General Health Questionnaire (GHQ), Profile of Mood States (POMS), Pittsburgh Sleep Quality Inventory (PSQI), Epworth Sleepiness Scale (ESS)). Results: The comparison between Group A and Group B showed significant differences after six months in C-reactive protein (*p* < 0.0001); after 12 months in aspartate aminotransferase (AST) (*p* < 0.0001) Viremia (*p* < 0.0001), HAI (*p* < 0.0012) and C-reactive protein (*p* < 0.0001); and at follow up in AST (*p* < 0.0001), Viremia (*p* < 0.0026) and C-reactive protein (*p* < 0.0001). Significant differences were observed after 12 month and follow-up in General Health Questionnaire, after 1, 6, 12 and follow-up in Profile of Mood States, after 6, 12, follow-up in Pittsburgh Sleep Quality Inventory and Epworth Sleepiness Scale. Conclusions: Supplementation with Resveratrol decreased General Health Questionnaire score and reduced sleep disorders in patients treated with Peg–IFN-α and RBV.

## 1. Background

Hepatitis C virus infection affects approximately 200 million individuals and contributes to increased mortality worldwide. The hepatitis C virus is characterized by a silent nature and long progression of liver disease. If left untreated, HCV infection puts patients at increased risk for cirrhosis and hepatocellular carcinoma [1,2,3,4,5].

HCV hepatitis may induce malaise, attention deficits, sleep disturbances, irritability, fatigue, chills, numbness and headaches, with high impact on quality of life and in work and social context.

Increasing the consumption of dietary fibers, flavonoids, phenol, anti-oxidant, micronutrients, carnitine, sylibin, and omega-3-polyunsaturated fatty acids is beneficial.

Resveratrol (RV) is a natural phenol found in red grapes, mulberries, peanuts, wines and tea, and can be extracted from red wine during fermentation of grape skin.

The natural source of resveratrol is *Polygonum cuspidatum*, a plant root extract which has been used in Oriental folk medicine [6,7,8,9].

RV shows anti-inflammatory, anti-oxidant and anti-cancer activities; prevents cardiovascular and cerebrovascular diseases; and reduces steatosis [10,11].

The mechanism by which RV exerts favorable effects is proposed to be related to induction of genes for oxidative phosphorylation and mitochondrial biogenesis.

Numerous data indicate that activation of a NAD^+^-dependent protein deacetylate Sirt1 is pivotal for RV action, and catalyzes deacetylation and activation of peroxisome proliferator gamma coactivator-1α, a cofactor in mitochondrial biogenesis [12,13].

Several potential causes have been proposed including direct invasion of the brain by the HCV virus, secondary inflammatory immune responses provoked by the presence of HCV in the central nervous system and comorbid medical and psychiatric factors observed in HCV populations [14].

Alternative therapies and dietary supplements are increasingly becoming commonplace [15,16,17,18].

In both in vitro and in vivo experiments, RV displays a wide range of beneficial effects in neurodegenerative and neurocognitive disorders [19]. Sockalingam Sanjeev et al. described sleep disorders common in hepatitis C patients and noted the paucity of research in this area [20]. Lang et al. surveyed 188 HCV naïve patients and found that sleeps problems were reported by about 65% [21]. Studies of Chronic Hepatitis C have shown that up to 97% of HCV patients endorse fatigue [22] and as many patients undergoing antiviral therapy with interferon endorse sleeping problems [23].

Recently, the importance of sleep problems, also expressed as disturbances of bedtime length, is considered as a possible reason reduction of quality of life in chronic hepatitis C [24].

The present study was designed to assess the impact of resveratrol on both the quality of sleep and disorders of sleep in a group of Hepatitis C patients treated with Peg-IFN-α and Ribavirin (RBV).

## 2. Patients and Methods

**Figure 1:**
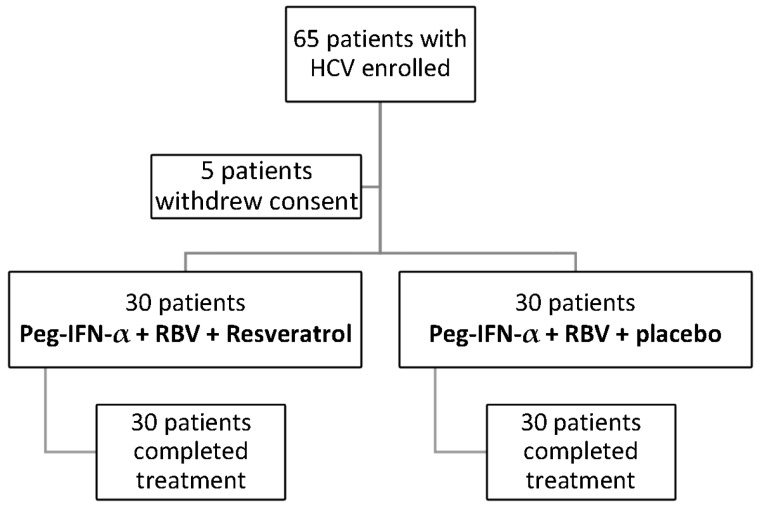
Profile of Peg-IFNα2b, RBV and Resveratrol treatment.

This observational study was conducted at the Department of Senescence, Cannizzaro Hospital, University of Catania (Italy), between February 2011 and July 2015. Sixty patients have been enrolled (35 males, 25 females) (Table 1). The patients received Peg-IFN-α-2b at dose 1.5 mg/kg per week; Ribavirin (RBV) at dose 1200 mg for 12 months; *N*-acetylcysteine 600 mg and lactoferrin 23.6 g three times day (Group A, 30 patients) or at dose 1.5 mg/kg per week; RBV at dose 1200 mg for 12 months; and *N*-acetylcysteine 600 mg, lactoferrin 23.6 g and RV in pills 19.8 mg three times day (Group B, 30 patients) for 12 months (Figure 1). The RV dosage was determined by studies on the best antioxidant and anti-inflammatory effect, improving also mitochondrial functions [25,26]. Patients were randomized into two groups (treatment versus placebo) using permuted-block randomization with an allocation ratio of 1:1 and a block size of 4. Random numbers were assigned to patients according to the sequence of their inclusion and patients received respective study products. Both clinical investigators and patients were blind to the product given. Peg-IFN-α, RBV and placebo were administered to subjects in Group A (Figure 1 trial profile). Peg-IFN-α, RBV, *N*-acetylcysteine 600 mg, lactoferrin 23.6 g and RV 19.8 mg were administered to subjects in Group B. Subjects were evaluated before starting therapy, and after 1, 6 and 12 months. Eligible patients were were 18 years of age or older, and HCV patients treated with Peg-Interferon and RBV. HCV infected populations must have elevated serum alanine transaminase levels and findings on liver biopsy consistent with chronic infection. Ineligible patients were those who had other liver diseases, positivity tests for serum HBsAg, for HIV antibodies, negativity for HCV antibodies and those who were affected by alcoholic liver disease, pulmonary and renal diseases, decompensated cirrhosis, pregnancy, hemoglobinopathies, autoimmune disorder, endocrinopathy and severe neuropsychiatric diseases. Hematochemical, virological, instrumental and histological analysis were performed on these patients. All subjects underwent a physical examination and medical interview before treatment. All patients were recruited in observation and respect of Helsinki Declaration [27] and gave their informed consent for the study participation and for each procedure they underwent. This study was approved by Cannizzaro Hospital Ethics Committee. All sensitive data were collected and protected in respect of present privacy statements.

### 2.1. Serum Analysis

All patients underwent a complete virological assay for HBV, HCV and anti-Delta (Delta virus antibody). Anti-HCV antibodies were determined by Enzyme-Linked immunosorbent assay (ELISA assay—Ortho Diagnostic Systems, Raritan, NJ, USA). HCV-RNA (Hepatitis C Virus RNA) levels were detected by polymerase chain reaction using HCV-RNA assay AmpliPrep/COBAS TaqMan (Roche Diagnostics Systems, Branchburg, NJ, USA). Serum samples negative for HCV RNA were retested using the Abbot Real Time HCV assay, with a lower limit of quantification and detection of 12 IU/mL HCV genotypes and subtypes were identified [28]. HCV viral genotypes were determined by restriction analysis of HCV-RNA 5″ UTR [29]. Aspartate Aminotransferase (AST) and Alanine Aminotransferase (ALT); gamma Glutamil Tranferase (γGT); total, conjugated and unconjugated bilirubin; and serum proteins analysis, as well as hematochemical measurements and virological analysis have been executed in the laboratory of Cannizzaro hospital with automated and standardized methods.

### 2.2. Histological Grading Assessment

Patients underwent ultrasound-assisted percutaneous biopsy [30]: tissue specimens were obtained with Menghini modified needles (Automatic Aspiration Needle for Liver Biopsy, ACR 16 G, 11 cm, manufactured by Sterylab Srl, Milan, Italy). A biopsy was considered adequate for evaluation if the specimen was >1.5 cm long and contained a minimum of 6 portal tracts. Knodell and Ishak Histological activity index (HAI) score was used to assess the histological grading of the disease [31]. The METAVIR scoring system was used to stage liver fibrosis as follows: F0, no fibrosis; F1, portal fibrosis without septa; F2, portal fibrosis and few septa; F3, numerous septa without cirrhosis; and F4, cirrhosis [32].

### 2.3. General Health Questionnaire (GHQ)

GHQ was the twenty-eight scaled version, which assesses somatic symptoms, anxiety and insomnia, social dysfunction and severe depression.

The twenty-eight items are scored from 0 to 3, with participants indicating the frequency or extent to which they have experienced a number of issues.

The items combine to assess the four sub-scales and the total scores ranges from 0 to 84, with higher scores representing more negative symptoms [33].

### 2.4. Profile of Mood States (POMS)

The POMS is a questionnaire of mood states and their functions in both clinical and research settings.

Participants rated sixty-five adjectives in terms of how much they had felt each one in the past week, using a five-point scale from “not at all” to “extremely”.

Scores from these sixty-five items (which includes seven dummy adjectives) are combined to give six global scores of “tension, depression, anger, fatigue, confusion and vigor”.

A total mood disturbance score can also be calculated by adding the scores from the first five of these global scores and subtracting “vigor” [34].

### 2.5. Pittsburgh Sleep Quality Inventory (PSQI)

The PSQI is a self-rating questionnaire, resulting in a global score between 0 and 21. The PSQI measure the quality and pattern of sleep. The PSQI assesses seven factors—subjective sleep quality, sleep latency, sleep duration, habitual sleep efficiency, sleep disturbances, use of sleep medication and daytime dysfunction—via questions regarding sleep timings on scales from 0 to 3, in which participants rate whether they have experienced a number of issues. A global sleep score is created by totaling the seven subfactors scores, with higher scores indicating poorer sleep quality [35].

### 2.6. Epworth Sleepiness Scale (ESS)

The ESS is a scale that measures daytime sleepiness.

The questionnaire asks the subject to rate his or her probability of falling asleep on a scale of increasing probability from 0 to 3 for eight different situations that people engage in during their daily lives.

The scores for the eight questions are added together to obtain a single number. Scores of 0–9 is considered normal. Scores of 10–15 indicate the possibility of mild to moderate sleep apnea. Score of 16 indicate severe sleep apnea [36].

### 2.7. Efficacy and Safety Assessment

We considered sustained virological responders (SVR) the patients with undetectable HCV-RNA 12 weeks after the end of therapy. The relapsers are the patients with as undetectable HCV-RNA levels at the end of treatment but detectable levels during the follow-up period. Patients with HCV-RNA detectable in the serum at the end of the treatment have been defined as Non Responders (NR). Reasons for discontinuation of the treatment were severe adverse events and absence of compliance.

### 2.8. Statistical Analysis

Data are expressed as means ± standard deviations (SD), median or percentage. Differences between groups were tested using Student’s *t*-test or Mann–Whitney U test and the Chi-square test was used for differences in the distribution of categorical variables. For sample size determination (power = 90%, alpha = 0.05), a drop-out rate of 20% was assumed and yielded a sample size of 60 patients in total. In all analyses, *p*-values < 0.05 were considered statistically significant. All statistical analyses were performed using SPSS 15.0 (SPSS Inc., Chicago, IL, USA).

## 3. Results

Demographic characteristics did not show significant differences between the two groups at baseline. The most frequent viral genotype was 1b (Table 1).

### 3.1. Effect of Resveratrol on Transaminases, Viremia and HAI 

In Group A, there was a significant decrease in AST (*p* < 0.001) and ALT (*p* < 0.001) after 12 months, and at follow up. Viremia was significantly reduced after 6 months (*p* < 0.05), 12 months (*p* < 0.001) (Table 2). HAI score decreased after 12 months (*p* < 0.001).

In the group that was treated with Peg-IFNα, RBV and RV, we observed a significant decrease in AST (*p* < 0.001), ALT (*p* < 0.001), and CRP (*p* < 0.001) after 6 and 12 months, and at follow up; a significant decrease we observed in viremia after 12 months and at follow-up. A significant decrease in HAI score (*p* < 0.001) was observed after 12 months. The comparison between Group A and Group B showed a significant difference after six months in ALT (*p* < 0.001), CRP (*p* < 0.001) and viremia (*p* < 0.05); and after 12 months in CRP (*p* < 0.001), ALT (*p* < 0.001), and AST (*p* < 0.001). At follow up, we observed a significant difference in AST (*p* < 0.05), ALT (*p* < 0.001) and CRP (*p* < 0.001).

In Group A, GHQ score increased after 1, 6, and 12 months, and at follow up (*p* < 0.05). POMS, PSQI and ESS score increased after 6 and 12 months, and at follow up (*p* < 0.05) (Table 3). In Group B, after one month, we observed a significant increase in GHQ (*p* < 0.001), POMS (*p* < 0.001) and PSQI (*p* < 0.001). The scores for POMS and PSQI decreased significantly after 12 months (*p* < 0.001) and at follow up (*p* < 0.05). The comparison between Group A and Group B showed a significant difference after 12 months in GHQ and POMS (*p* < 0.001), and PSQI and ESS (*p* < 0.05); and after 12 months in ESS (*p* < 0.05).

### 3.2. Comparison between Patients According to Response Treatment

According to the response treatment, in subjects treated with Peg IFN-α + RBV alone, comparing pre-treatment and SVR patients, there was a significant decrease in POMS (*p* < 0.05) and in ESS (*p* < 0.001), while, in the comparison between pre-treatment and non responders, we observed an increase in PSQI (*p* < 0.001) . In subjects treated with Peg IFN-α + RBV + RV, we observed a decrease in POMS, PSQI and ESS (*p* < 0.01) in SVR patients. Lastly, in the comparison between treated with RV and without RV, we have observed a decrease in GHQ, POMS, PSQI and ESS (*p* < 0.01) (Table 4).

## 4. Adverse Events

No serious adverse events (WHO grade 3 or 4) have been observed in both groups.

Twenty patients showed mild psychological disorders such as anxiety, irritability, fatigue, and depression. Noteworthy a higher increase of these disorders was observed in the Peg-IFN-α + RBV alone treatment (Table 5).

## 5. Discussion

Sleep disorders represent a very common problem among HCV patients treated with Peg-IFN-α and RBV; sleep disorders were recognized in nearly three-quarters of the analyzed group.

Polyphenolic natural products alter cellular metabolism, which has great impacts on cellular inflammatory status and function [37].

HCV and IFN treatment have shown to be elevated risk factors for depression, irritability, anxiety and sleep disorders. Our efforts in HCV treatment have been focused on understanding the impact of HCV infection on sleep disturbances and general health [38].

Sleep disturbances may exacerbate chronic fatigue, which can occur in up to 70% of HCV patients, and reduce health-related quality of life [39,40,41,42].

Few studies have been carried out to date to show a relationship between sleep disturbance, hepatitis C and IFN treatment. In our study we observed in HCV patients treated with Peg-IFN-α and RBV alone an increase of somatic symptoms, anxiety, depression, insomnia, mood dysfunction, social dysfunction, anger, fatigue, confusion, sleep apnea and poor sleep quality. The majority of results show sleep disturbances in HCV patients treated with Peg-IFN and RBV at one month and at six months following treatment. Sleep disturbances were significantly associated with various phases of treatment, and more physical and neuropsychological symptoms. It is plausible that inflammatory cytokines serve as mediators of both environmental and genetic factors that may trigger the development and progression of anxiolytic and depressive disorders.

The cytokines may induce depression by crossing the blood–brain barrier, and activating neuroendocrine and neurotransmitter systems. In fact, IFN-α has been shown to increase degradation of 5-hidroxytryptamine (5-HT) and tryptophan. IFN-α also decreases mRNA levels and protein expression of 5-HT receptor 1A, resulting in down-regulation and thus lower concentration of 5HT [43,44].

Supplementation with silybin, carnitine and its derivate, rosuvastatin, reduces adverse effects and improves quality of life [40].

In the comparison of the two study groups, we observed not only a decreased of general health, but also a decreased sleep quality and daytime sleepiness in HCV peg-IFN-α and RBV alone.

Neuropsychiatric sequel of IFN treatment can include sleep disturbances, which may be a harbinger for IFN-α induced disorders.

The protective effect of RV may be attributed to its antioxidant and free radicals scavenging properties [45,46].

In patients treated with peg-IFN-α, RBV and RV, we observed lower scores and reduced sleep disturbance. The reduction in anxiety, depression and sleep disturbance symptoms associated with RV supplementation provides the first evidence that RV may have potential anxiolytic benefits for individuals. Accordingly, alterations in inflammation could also influence anxiety.

Recent studies have demonstrated that pro-inflammatory cytokines play a relevant role in the regulation and modulation of sleep and contribute to the physiological control of the sleep-wake cycle [47,48].

**Figure 2:**
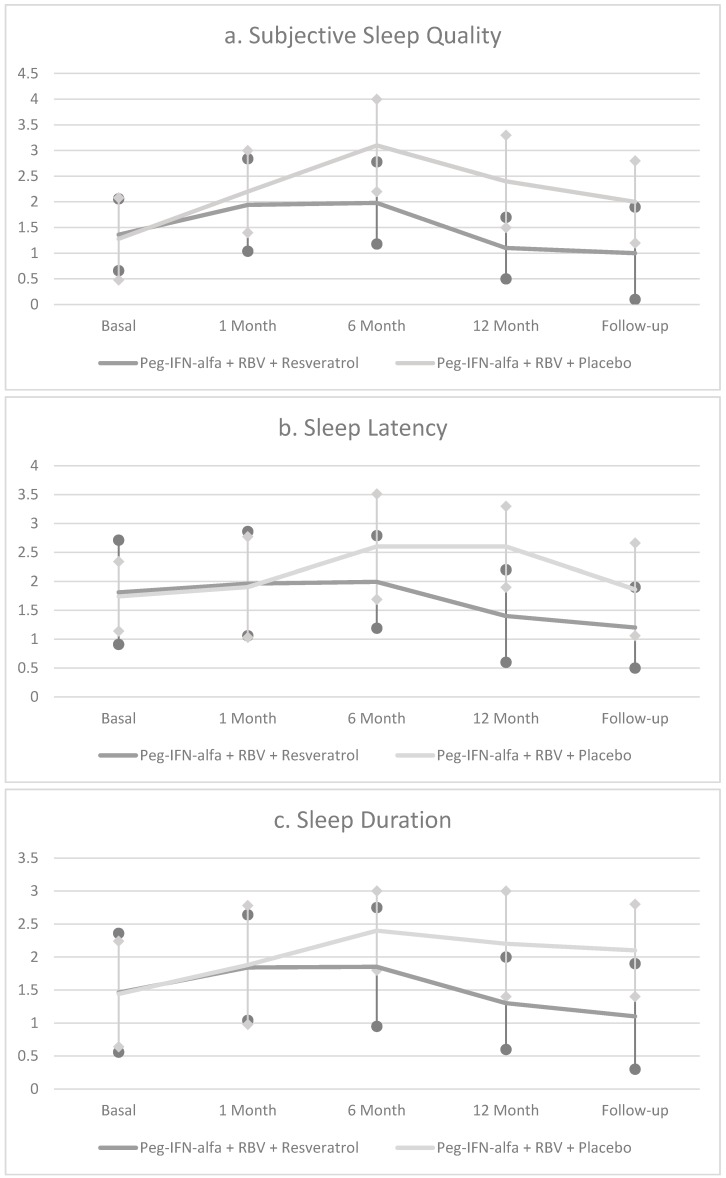

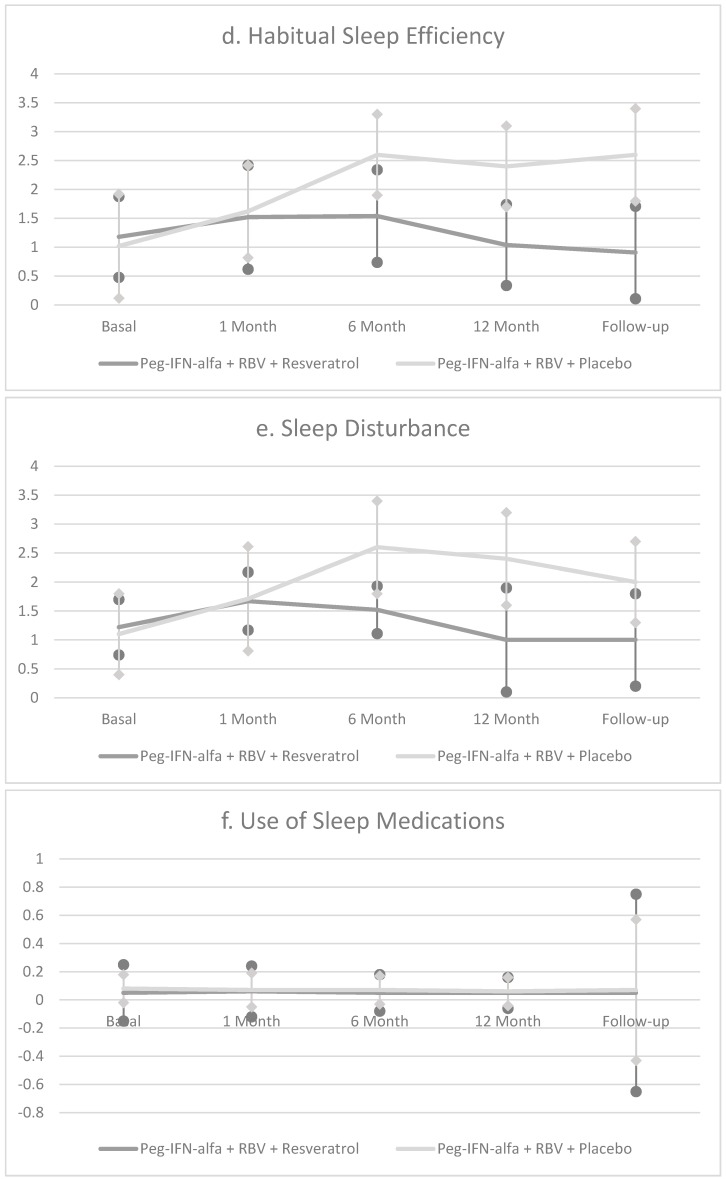

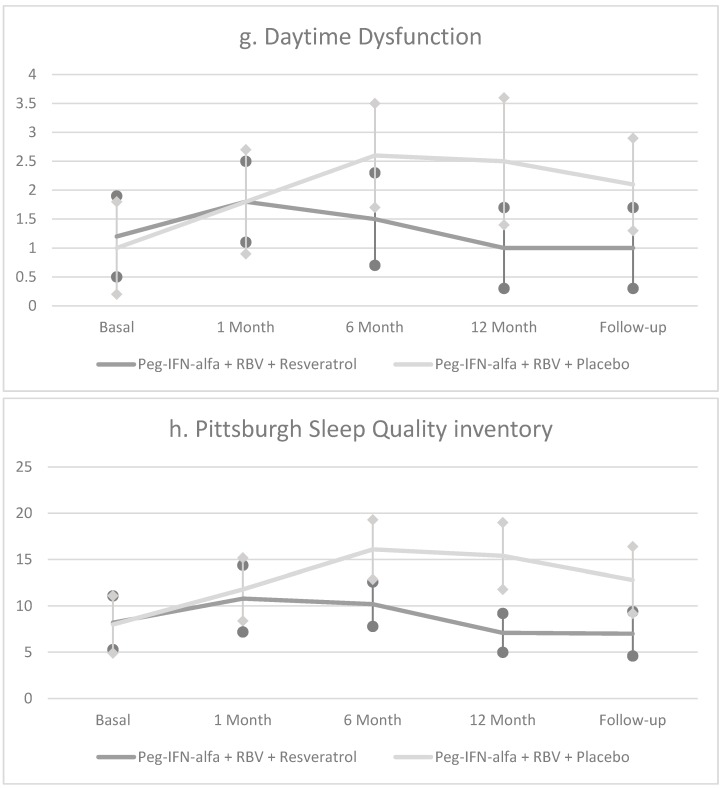
Representation of PSQI subfactors scores at baseline, and after 1, 6 and 12 months (mean ± SD) in HCV patients treated with Peg-IFN and Ribavirin with and without Resveratrol.

These data showed that RV supplementation benefited clinically depressed individuals, but not those with less severe depressed mood. AST, ALT, γGT, CRP, TNF-α and IL-6 as well as the histological characteristics (steatosis and fibrosis of the patients) were significantly improved compared with the patients who received the placebo. Supplementation with RV increased the sustained virological response (SVR) in patients treated with Peg-IFN-α and RBV. Several studies suggested poor sleep quality and sleep disturbances, which are associated with subsequent depression during IFN therapy. According to response treatment, in both HCV patients treated with Peg IFN-α + RBV alone and Peg IFN-α + RBV + RV, RV improved sleep quality. The beneficial effects on sleep disturbance were observed in all patients, both SVR and NR, treated with RV compared with the SVR and NR patients that were treated with Peg INF-α and Ribaverin alone (Figure 2).

A human intervention study indicated that consumption of RV increased the number of *Enterococcus*, *Prevotella*, and *Bacterioides uniformis*, while the quantity of *Lactobacillus* spp. was unaltered [49].

Individuals with higher anxiety traits, depression, and fatigue showed a distinct metabolic profile, indicative of a different energy homeostasis, hormonal metabolism and gut microbial activity [50,51]. The daily consumption of polyphenols resulted in a significant modification in the microbial metabolism, with potential long-term health consequences.

Little research exists regarding the effect of polyphenols on mood but this anti-fatigue effect may find an explanation in vitro and in animal studies, which report the ability of RV to inhibit activity of monoamine oxidase A/B.

Red wine polyphenol shows anti-fatigue activity through increased energy expenditure and endurance capacity in mice [12].

The limitations of this study include the small number of patients, who were not stratified by disease severity, and not including all patients with chronic liver diseases. Another limitation is that comparisons were made between a real disease that these patients were suffering from, and perceived rather than real losses.

## 6. Conclusions

This study adds weight to the argument that HCV patients should be treated, not only to improve disease prognosis, but also to potentially improve quality of life.

Intervention targeting the psychological, social and economic circumstances experienced by HCV patients and its treatment may be able to influence health status.

Further research is essential to determine whether some component of the psychosocial health burden associated with HCV infection and its treatment can be mitigated.

Future studies are needed to explore factors related to maintain the sleep quality as well as physical and psychological disablement and quality of life.

## Figures and Tables

**Table 1:** Patients characteristics at liver biopsy.

Parameter	Group A 30 pt (Peg-IFN-α + RBV + Placebo)	Group B 30 pt (Peg-IFN-α + RBV + Resveratrol)	*p*-Value
Male	18	17	NS
Female	12	13	NS
Route of transmission of HCV (No. of patients)			
Blood transfusion	16	12	NS
Intravenous drug abuse	3	5	NS
Occupational	1	3	NS
Unknown	11	11	NS
HCV genotype			
1a	1	2	NS
1b	23	23	NS
2a	3	3	NS
3a	1	2	NS
Mean age (years)	46.8 ± 4.4	46.4 ± 4.1	NS
HCV exposure time (years)	3.87 ± 3.4	3.91 ± 3.8	NS
Body Mass Index (BMI) (kg/m^2^)	25.8 ± 3.4	25.4 ± 3.6	NS
Plasma glucose (mmol/L) (normal 3.9–6.4)	5.4 ± 0.74	6.3 ± 0.71	NS
Aspartate Aminotrasferase (AST) (IU/L) (normal 15–50)	143.4 ± 33.2	162.4 ± 32.1	NS
Alanine Aminotrasferase (ALT) (IU/L) (normal 15–50)	155.4 ± 36.1	162.4 ± 32.1	NS
Viremia (10^6^ copies/mL)	3.4 ± 2.1	3.7 ± 2.8	NS
C-Reactive Protein (CRP) (mg/dL) (normal < 1.0)	6.4 ± 0.9	6.5 ± 0.8	NS
HAI	10.4 ± 2.8	10.5 ± 2.2	NS

**Table 2:** Characteristics of subjects at baseline, after 6 and 12 months, and at follow-up. Values are expressed as Mean ± SD.

	Group A Peg-IFN α + RBV + Placebo (*n* = 30)				Group B Peg-IFN α + RBV + Resveratrol (*n* = 30)			
Parameters	Before Treatment	After 6 Months	After 12 Months	Follow-Up	Before Treatment	After 6 Months	After 12 Months	Follow-Up
Aspartate Aminotransferase (AST) (IU/L)	143.4 ± 33.2	107.1 ± 34.6 ^A^***	74.2 ± 22.1 ^C^***	66 ± 22.4 ^C^***	144 ± 33.8	96.1 ± 35.6 ^A^***	51.6 ± 14.2 ^C^***	44.2 ± 16.2 ^C^***
Alanine Aminotransferase (ALT) (IU/L)	155.4 ± 36.1	122 ± 31.4 ^A^***	68.1 ± 16.4 ^A^***	64.1 ± 18.2 ^A^***	162.4 ± 2.8	104.1 ± 36.4 ^A^***	61.8 ± 13.2 ^A^***	56.2 ± 14.4 ^A^***
Bilirubin (mmol/L)	10.5 ± 7.1	10.2 ± 7.3 ^A^*	10.2 ± 6.6 ^A^*	10.2 ± 6.8 ^A^*	10.5 ± 4.9	10.1 ± 4.8 ^A^*	10.2 ± 3.0 ^A^*	10.3 ± 3.6 ^A^*
Albumin (g/dL)	4.2 ± 0.7	4.2 ± 0.94 ^A^*	4.1 ± 0.9 ^A^*	4.1 ± 0.7 ^A^*	4.2 ± 0.6	4.3 ± 0.7 ^A^*	4.2 ± 0.9 ^A^*	4.1 ± 0.7 ^A^*
Viremia (106 copies/mL)	3.4 ± 2.1	3.3 ± 2.4 ^A^*	2.96 ± 0.9 ^C^*	2.98 ± 0.8 ^C^*	3.7 ± 2.14	3.1 ± 2.5 ^A^*	1.8 ± 1.2 ^C^***	1.9 ± 1.7 ^C^***
HAI	10.4 ± 2.8	-	9.7 ± 2.4 ^C^*	-	10.9 ± 3.4	-	8.1 ± 0.9 ^C^***	-
C-Reactive Protein (CRP)	6.4 ± 0.9	6.0 ± 0.8 ^C^*	5.9 ± 0.8 ^C^**	5.4 ± 0.7 ^C^***	6.5 ± 0.8	5.2 ± 0.7 ^C^***	3.5 ± 0.8 ^C^***	3.8 ± 0.7 ^C^***

Comparison within groups: * NS; ** *p* < 0.05; *** *p* < 0.001; Comparison between groups: ^A^ NS; ^B^ *p* < 0.05; ^C^ *p* < 0.001; There were no significant differences between groups at baseline.

**Table 3:** Scores of General Healt Questionnaire (GHQ), Profile of Mood States (POMS), Pittsburgh Sleep Quality Inventory (PSQI) and Epworth Sleepiness Scale (ESS) in the study groups. Values are expressed as Mean ± SD.

	Before Treatment	After 1 Month	After 6 Months	After 12 Months	Follow-Up
**Group A Peg-IFN α + RBV + Placebo (*n* = 30)**					
General Health Questionnaire (GHQ)	28.1 ± 12.4	52.1 ± 14.1 ^A^***	48.2 ± 14.4 ^A^***	42.3 ± 13.4 ^C^***	40.2 ± 14.4 ^B^***
Profile of Mood States (POMS)	184.2 ± 13.7	290 ± 13.8 ^C^***	260.1 ± 13.9 ^C^***	256.4 ± 14.1 ^C^***	250.1 ± 10.8 ^C^***
Pittsburgh Sleep Quality Inventory (PSQI)	8.0 ± 3.1	11.8 ± 3.4 ^A^***	16.1 ± 3.2 ^C^***	15.4 ± 3.6 ^C^***	12.8 ± 3.6 ^C^***
Epworth Sleepiness Scale (ESS)	12.1 ± 2.2	16.4 ± 3.0 ^A^***	15.4 ± 3.0 ^B^***	12.9 ± 3.0 ^B^*	12.2 ± 2.4 ^B^*
**Group B Peg-IFN-α + RBV + Resveratrol (*n* = 30)**					
General Health Questionnaire (GHQ)	29.1 ± 16.1	48.1 ± 13.2 ^A^***	44.2 ± 15.2 ^A^***	30.4 ± 12.8 ^C^*	30.2 ± 12.6 ^B^*
Profile of Mood States (POMS)	187.2 ± 12.4	208.4 ± 13.2 ^C^***	191 ± 13.8 ^C^*	170 ± 12.6 ^C^***	168 ± 12.9 ^C^***
Pittsburgh Sleep Quality Inventory (PSQI)	8.2 ± 2.9	10.8 ± 3.6 ^A^**	10.2 ± 2.4 ^C^**	7.1 ± 2.1 ^C^*	7.0 ± 2.4 ^C^*
Epworth Sleepiness Scale (ESS)	12.8 ± 2.4	16.4 ± 3.2 ^A^***	13.4 ± 3.1 ^B^*	10.4 ± 2.7 ^B^**	10.6 ± 2.8 ^B^**

Comparison within groups: * NS; ** *p* < 0.05; *** *p* < 0.001; Comparison between groups: ^A^ NS; ^B^ *p* < 0.05; ^C^ *p* < 0.001; There were no significant differences between groups at baseline.

**Table 4:** Comparison between patients according to the response treatment.

	Group A 30 pt (Peg-IFN-α + RBV + Placebo)			Group B 30 pt (Peg-IFN-α + RBV + Resveratrol)		
	Pretreatment	SVR	Non R	Pretreatment	SVR	Non R
		**14**	**16**		**19**	**11**
General Health Questionnaire (GHQ)	28.1 ± 12.4	26.7 ± 10.9 °^A^*	29.7 ± 11.8 °^A^*	29.1 ± 16.1	20.8 ± 14.2 °°^A^*	32.8 ± 14.9 °°^A^*
Profile of Mood States (POMS)	184.2 ± 13.7	175.1 ± 12.0 °^A^**	180.0 ± 13.8 °^B^*	187.2 ± 12.4	174.4 ± 11.8 ^°°°A^***	194.0 ± 12.1 ^°°°B^*
Pittsburgh Sleep Quality Inventory (PSQI)	8.0 ± 3.1	7.5 ± 2.4 °°°^A^*	11.0 ± 2.5 °°°^A^**	8.2 ± 2.9	6.0 ± 2.1 °°°^A^*	10.8 ± 2.4 °°°^A^**
Epworth Sleepiness Scale (ESS)	12.1 ± 2.2	9.2 ± 2.3 °°^B^***	12.0 ± 2.0 °°^A^*	12.8 ± 2.4	7.4 ± 2.2 °°°^B^***	11.9 ± 2.1 °°°^A^*

SVR: Sustained Viral Response; Non R: Non Responders; Comparison within groups: * NS; ** *p* < 0.05; *** *p* < 0.001; Comparison SVR-Non R within groups: ° NS; °° *p* < 0.05; °°° *p* < 0.001; Comparison between groups: ^A^ NS; ^B^ *p* < 0.05; ^C^ *p* < 0.001; There were no significant differences between groups at baseline.

**Table 5:** Adverse events observed in the study population.

	Group A (*n* = 30) (Peg-IFNa + RBV + Placebo)	Group B (*n* = 30) (Peg-IFNa + RBV + Resveratrol)
Psychological disorders	18%	15%
Hypercholesterolemia	16%	20%
Fatigue	48%	44%
Headache	36%	30%
Musculoskeletal pain	51%	28%
Myalgia	44%	32%
Hypertriglyceridemia	48%	27%
Nausea	15%	18%
Anorexia	10%	12%
Irritability	22%	18%
Hyperglycemia	15%	7%
Weight loss	13%	14%
Decrease of hemoglobin values at the end of treatment	from 13.4 g/dL (range 11.4–14.4) to 11.4 g/dL (range 10.4–14.0 g/dL)	from 13.5 g/dL (range 11.6–15.3 g/dL) to 10.6 (range 10.4–13.8 g/dL)

## References

[1] Smith B.D., Morgan R.L., Beckett G.A., Falck-Ytter Y., Holtzman D., Teo C.-G., Alter M. (2012). Recommendations for the identification of chronic hepatitis C virus infection among persons born during 1945–1965. Morb. Mortal. Wkly. Rep. Recomm. Rep..

[2] Hajarizadeh B., Grebely J., Dore G.J. (2013). Epidemiology and natural history of HCV infection. Nat. Rev. Gastroenterol. Hepatol..

[3] Bertino G., Ardiri A., Proiti M., Rigano G., Frazzetto E., Demma S., Rapisarda V. (2016). Chronic hepatitis C: This and the new era of treatment. World J. Hepatol..

[4] Malaguarnera G., Catania V.E., Francaviglia A., Malaguarnera M., Drago F., Motta M., Latteri S. (2017). Lipoprotein (a) in patients with hepatocellular carcinoma and portal vein thrombosis. Aging Clin. Exp. Res..

[5] Hilsabeck R.C., Hassanein T.I., Carlson M.D., Ziegler E.A., Perry W. (2003). Cognitive functioning and psychiatric symptomatology in patients with chronic hepatitis C. J. Int. Neuropsychol. Soc..

[6] Malaguarnera M., Motta M., Vacante M., Malaguarnera G., Caraci F., Nunnari G., Bertino G. (2015). Silybin-vitamin E-phospholipids complex reduces liver fibrosis in patients with chronic hepatitis C treated with pegylated interferon α and ribavirin. Am. J. Transl. Res..

[7] Calland N., Sahuc M.-E., Belouzard S., Pène V., Bonnafous P., Mesalam A.A., Lambert O. (2015). Polyphenols Inhibit Hepatitis C Virus Entry by a New Mechanism of Action. J. Virol..

[8] Grosso G., Godos J., Lamuela-Raventos R., Ray S., Micek A., Pajak A., Galvano F. (2017). A comprehensive meta-analysis on dietary flavonoid and lignan intake and cancer risk: Level of evidence and limitations. Mol. Nutr. Food Res..

[9] Grosso G., Micek A., Godos J., Pajak A., Sciacca S., Galvano F., Giovannucci E.L. (2017). Dietary Flavonoid and Lignan Intake and Mortality in Prospective Cohort Studies: Systematic Review and Dose-Response Meta-Analysis. Am. J. Epidemiol..

[10] Heebøll S., Thomsen K.L., Pedersen S.B., Vilstrup H., George J., Grønbæk H. (2014). Effects of resveratrol in experimental and clinical non-alcoholic fatty liver disease. World J. Hepatol..

[11] Bhullar K.S., Hubbard B.P. (2015). Lifespan and healthspan extension by resveratrol. Biochim. Biophys. Acta.

[12] Lagouge M., Argmann C., Gerhart-Hines Z., Meziane H., Lerin C., Daussin F., Geny B. (2006). Resveratrol improves mitochondrial function and protects against metabolic disease by activating SIRT1 and PGC-1alpha. Cell.

[13] Cunningham J.T., Rodgers J.T., Arlow D.H., Vazquez F., Mootha V.K., Puigserver P. (2007). mTOR controls mitochondrial oxidative function through a YY1-PGC-1alpha transcriptional complex. Nature.

[14] Vargas H.E., Laskus T., Radkowski M., Wilkinson J., Balan V., Douglas D.D., Harrison M.E., Mulligan D.C., Olden K., Adair D. (2002). Detection of hepatitis C virus sequences in brain tissue obtained in recurrent hepatitis C after liver transplantation. Liver Transpl..

[15] Malaguarnera G., Bertino G., Chisari G., Motta M., Vecchio M., Vacante M., Caraci F., Greco C., Drago F., Nunnari G. (2016). Silybin supplementation during HCV therapy with pegylated interferon-α plus ribavirin reduces depression and anxiety and increases work ability. BMC Psychiatry.

[16] Malaguarnera G., Pennisi M., Gagliano C., Vacante M., Malaguarnera M., Salomone S., Drago F., Bertino G., Caraci F., Nunnari G. (2014). Acetyl-l-carnitine supplementation during HCV therapy with pegylated interferon-α 2b plus ribavirin: Effect on work performance; a randomized clinical trial. Hepat. Mon..

[17] Vecchio M., Gracies J.-M., Panza F., Fortunato F., Vitaliti G., Malaguarnera G., Santamato A. (2017). Change in Coefficient of Fatigability Following Rapid, Repetitive Movement Training in Post-Stroke Spastic Paresis: A Prospective Open-Label Observational Study. J. Stroke Cerebrovasc. Dis..

[18] Malaguarnera M. (2013). Acetyl-l-carnitine in hepatic encephalopathy. Metab. Brain Dis..

[19] Park S.J., Ahmad F., Philp A., Baar K., Williams T., Luo H., Kim M.K. (2012). Resveratrol ameliorates aging-related metabolic phenotypes by inhibiting cAMP phosphodiesterases. Cell.

[20] Sockalingam S., Abbey S.E., Alosaimi F., Novak M. (2010). A review of sleep disturbance in hepatitis C. J. Clin. Gastroenterol..

[21] Lang C.A., Conrad S., Garrett L., Battistutta D., Cooksley W.G., Dunne M.P., Macdonald G.A. (2006). Symptom prevalence and clustering of symptoms in people living with chronic hepatitis C infection. J. Pain Symptom Manag..

[22] Poynard T., Cacoub P., Ratziu V., Myers R.P., Dezailles M.H., Mercadier A., Ghillani P., Charlotte F., Piette J.C., Moussalli J. (2002). Fatigue in patients with chronic hepatitis C. J. Viral Hepat..

[23] Lotrich F.E., Ferrell R.E., Rabinovitz M., Pollock B.G. (2009). Risk for depression during interferon-alpha treatment is affected by the serotonin transporter polymorphism. Biol. Psychiatry.

[24] Malaguarnera G., Vacante M., Drago F., Bertino G., Motta M., Giordano M., Malaguarnera M. (2015). Endozepine-4 levels are increased in hepatic coma. World J. Gastroenterol..

[25] Kim S., Jin Y., Choi Y., Park T. (2011). Resveratrol exerts anti-obesity effects via mechanisms involving down-regulation of adipogenic and inflammatory processes in mice. Biochem. Pharmacol..

[26] Davinelli S., Scapagnini G., Marzatico F., Nobile V., Ferrara N., Corbi G. (2017). Influence of equol and resveratrol supplementation on health-related quality of life in menopausal women: A randomized, placebo-controlled study. Maturitas.

[27] (1967). Declaration of Helsinki. Recommendations guiding doctors in clinical research. Adopted by the World Medical Association in 1964. Wis. Med. J..

[28] Stuyver L., Wyseur A., van Arnhem W., Hernandez F., Maertens G. (1996). Second-generation line probe assay for hepatitis C virus genotyping. J. Clin. Microbiol..

[29] Simmonds P., Alberti A., Alter H.J., Bonino F., Bradley D.W., Brechot C., Brouwer J.T., Chan S.W., Chayama K., Chen D.S. (1994). A proposed system for the nomenclature of hepatitis C viral genotypes. Hepatology.

[30] Latteri S., Malaguarnera G., Mannino M., Pesce A., Currò G., Tamburrini S., Scuderi M. (2017). Ultrasound as point of care in management of polytrauma and its complication. J. Ultrasound.

[31] Knodell R.G., Ishak K.G., Black W.C., Chen T.S., Craig R., Kaplowitz N., Kiernan T.W., Wollman J. (1981). Formulation and application of a numerical scoring system for assessing histological activity in asymptomatic chronic active hepatitis. Hepatology.

[32] Bedossa P., Poynard T. (1996). An algorithm for the grading of activity in chronic hepatitis C. The METAVIR Cooperative Study Group. Hepatology.

[33] Goldberg D. (1984). The Recognition of Psychiatric Illness by Non-Psychiatrists. Aust. N. Z. J. Psychiatry.

[34] McNair D.M., Lorr M., Droppleman L.F. (1971). Manual for the Profile of Mood States.

[35] Buysse D.J., Reynolds C.F., Monk T.H., Berman S.R., Kupfer D.J. (1989). The Pittsburgh sleep quality index: A new instrument for psychiatric practice and research. Psychiatry Res..

[36] Johns M.W. (1991). A new method for measuring daytime sleepiness: The Epworth sleepiness scale. Sleep.

[37] Carlson M.D., Hilsabeck R.C., Barakat F., Perry W. (2010). Role of Sleep Disturbance in Chronic Hepatitis C Infection. Curr. Hepat. Rep..

[38] Chisari G., Stagni E., Rampello L., Malaguarnera M., Chisari C.G. (2013). The ocular surface in Patients video display terminal (VDT). Acta Med. Mediterr..

[39] Kallman J., O’Neil M.M., Larive B., Boparai N., Calabrese L., Younossi Z.M. (2007). Fatigue and health-related quality of life (HRQL) in chronic hepatitis C virus infection. Dig. Dis. Sci..

[40] Malaguarnera M., Vacante M., Russo C., Gargante M.P., Giordano M., Bertino G., Volti G.L. (2011). Rosuvastatin reduces nonalcoholic fatty liver disease in patients with chronic hepatitis C treated with α-interferon and ribavirin. Hepat. Mon..

[41] Malaguarnera M., Vacante M., Condorelli G., Leggio F., Di Rosa M., Motta M., Malaguarnera G., Alessandria I., Rampello L., Chisari G. (2013). Probiotics and prebiotics in the management of constipation in the elderly. Acta Med. Mediterr..

[42] Malaguarnera M., Cristaldi E., Romano G., Malaguarnera L. (2012). Autoimmunity in the elderly: Implications for cancer. J. Cancer Res. Ther..

[43] Asara Y., Melis A., De Luca L.M., Bozzo C., Castiglia P., Chessa G., Marchal J.A. (2016). Influence of metals on rhinosinusal polyposis in Sardinian population (Italy). Environ. Sci. Pollut. Res. Int..

[44] Cai W., Khaoustov V.I., Xie Q., Pan T., Le W., Yoffe B. (2005). Interferon-alpha-induced modulation of glucocorticoid and serotonin receptors as a mechanism of depression. J. Hepatol..

[45] Xu Y., Wang Z., You W., Zhang X., Li S., Barish P.A., Ogle W.O. (2010). Antidepressant-like effect of trans-resveratrol: Involvement of serotonin and noradrenaline system. Eur. Neuropsychopharmacol. J. Eur. Coll. Neuropsychopharmacol..

[46] Queipo-Ortuño M.I., Boto-Ordóñez M., Murri M., Gomez-Zumaquero J.M., Clemente-Postigo M., Estruch R., Tinahones F.J. (2012). Influence of red wine polyphenols and ethanol on the gut microbiota ecology and biochemical biomarkers. Am. J. Clin. Nutr..

[47] Opp M.R. (2005). Cytokines and sleep. Sleep Med. Rev..

[48] Malaguarnera G., Bertino G., Greco C., Gagliano C., Motta M., Chisari G., Malaguarnera M. (2017). Job performance in chronic hepatitis C virus patients treated with pegylated interferon-α2b plus ribavirin: An observational study. Transl. Med. Commun..

[49] Imeri L., Opp M.R. (2009). How (and why) the immune system makes us sleep. Nat. Rev. Neurosci..

[50] Chisari G., Rampello L., Chisari E., Catania V., Greco C., Stagno E., Chisari C. (2016). Microbiology synergism between tear substitutes and symbiotic treatment of patients with irritable bowel syndrome. Acta Med. Mediterr..

[51] Beloborodova N., Bairamov I., Olenin A., Shubina V., Teplova V., Fedotcheva N. (2012). Effect of phenolic acids of microbial origin on production of reactive oxygen species in mitochondria and neutrophils. J. Biomed. Sci..

